# A Review on Multiple I-III-VI Quantum Dots: Preparation and Enhanced Luminescence Properties

**DOI:** 10.3390/ma16145039

**Published:** 2023-07-17

**Authors:** Ting Chen, Yuanhong Chen, Youpeng Li, Mengbiao Liang, Wenkui Wu, Yude Wang

**Affiliations:** 1 Institute of Materials Science & Devices, Suzhou University of Science and Technology, Suzhou 215009, China; chenyuanhong1997@163.com (Y.C.); liyoupeng1999@163.com (Y.L.); 17706248404@163.com (M.L.); 15890543902@163.com (W.W.); 2 National Center for International Research on Photoelectric and Energy Materials, School of Materials and Energy, Yunnan University, Kunming 650504, China

**Keywords:** multiple quantum dots, metal ion doping, surface coating, optoelectronic, white light emitting diodes

## Abstract

I-III-VI type QDs have unique optoelectronic properties such as low toxicity, tunable bandgaps, large Stokes shifts and a long photoluminescence lifetime, and their emission range can be continuously tuned in the visible to near-infrared light region by changing their chemical composition. Moreover, they can avoid the use of heavy metal elements such as Cd, Hg and Pb and highly toxic anions, i.e., Se, Te, P and As. These advantages make them promising candidates to replace traditional binary QDs in applications such as light-emitting diodes, solar cells, photodetectors, bioimaging fields, etc. Compared with binary QDs, multiple QDs contain many different types of metal ions. Therefore, the problem of different reaction rates between the metal ions arises, causing more defects inside the crystal and poor fluorescence properties of QDs, which can be effectively improved by doping metal ions (Zn^2+^, Mn^2+^ and Cu^+^) or surface coating. In this review, the luminous mechanism of I-III-VI type QDs based on their structure and composition is introduced. Meanwhile, we focus on the various synthesis methods and improvement strategies like metal ion doping and surface coating from recent years. The primary applications in the field of optoelectronics are also summarized. Finally, a perspective on the challenges and future perspectives of I-III-VI type QDs is proposed as well.

## 1. Introduction

Semiconductor quantum dots (QDs) are a kind of quasi-zero-dimensional material. When the particle size of QDs is close to or less than the exciton Bohr radius of the main material, they always show unique physical and chemical properties, i.e., a high photoluminescence quantum yield (PLQY) and tunable range of luminescent peaks due to the quantum domain effect and size effect [1,2]. All these advantages have made them potential candidates in the fields of bioimaging [3], white light-emitting diodes (WLEDs) [4], solar cells [5] and photocatalytic reductions [6,7] over the past thirty years. Although CdSe binary QDs have achieved rapid development in preparation methods and commercial applications, the carcinogenic Cd element easily causes serious human health and ecological problems, which restricts their large-scale commercial application. Moreover, no-Cd-based QDs (such as ZnSe [8], Ag_2_S [9], etc.) have been successfully prepared, but there are still many problems such as low PLQY and narrow luminous peak coverage, resulting in a low optical performance compared with Cd-based QDs, while InP QDs have achieved comparable luminescence properties with Cd-based QDs, but their preparation process is difficult and expensive [10,11,12]. Therefore, for scientific researchers, the exploration of green QDs with high PLQY and low cost has become a major problem to be solved urgently.

Recently, the multiple environmentally friendly I-III-VI type QDs, such as CuInS_2_, CuInSe_2_, CuGaS_2_, AgInS_2_, AgInSe_2_ and AgGaS_2_ QDs, show the advantages of environmental friendliness, wide emission spectrum coverage, good tunability, large Stokes shifts, long fluorescence life, anti-photobleaching stability, etc. [13,14]. Therefore, they are ideal materials to replace the traditional binary QDs for applications in photocatalytic reduction [6], WLEDs [4,7], solar cells [5] and bioimaging fields. In 2004, Castro et al. [15] first reported the preparation of CuInS_2_ QDs with an emission peak between 662 and 673 nm by pyrolysis of a single precursor (PPh_3_)_2_CuIn(SEt)_4_ at 200 °C. Dai et al. [16] synthesized non-stoichiometric AgInS_2_ QDs by the thermal decomposition of single-source precursors in a solution of two kinds of primary amines (oleylamine and octylamine). By changing the chemical composition of the precursor, the content of Ag^+^ in the QDs can be regulated, so that non-stoichiometric AgInS_2_ QDs with a large number of Ag vacancies can be prepared. When the molar ratio of Ag^+^ to total metal ions varies between 0.1 and 0.7, the absorption initiation wavelength range can be adjusted from 750 to 580 nm and gradually shifts blue with the decrease of Ag^+^ content. This experiment effectively proves that the unique advantage that the optical properties of I-III-VI type QDs can be regulated by changing the chemical composition. By developing new synthetic methods and adjusting the experimental conditions rationally, the precise regulation of the material phase, morphology and photoelectric properties of I-III-VI type QDs can be realized. Moreover, their luminous peaks can achieve almost full spectral coverage. Therefore, they have shown extremely broad application prospects in the field of optoelectronics [17,18]. However, at present, most high-quality I-III-VI type QDs are obtained through organic phase synthesis processes, which always require harsh experimental conditions, such as an inert atmosphere, high synthesis temperature, etc. Meanwhile, the use of a large number of organic solvents increases the cost and toxicity for commercial applications. Because the surfaces of QDs are modified by lipophilic groups, the QDs cannot be directly applied in an aqueous phase environment. Although the ligand exchange provides a viable way for the hydrophilic transformation of QDs, the tedious exchange steps as well as the large decay of fluorescence properties greatly restrict its application [19,20]. Therefore, the preparation of I-III-VI QDs in an aqueous phase environment has been attracting more and more attention in recent years [21,22,23]. In addition, the fluorescence intensity and stability of multiple QDs still need to be improved because of the different reactivity of various cations contained in multiple QDs, which makes the internal defects of the crystal more numerous. Coating with shell materials, i.e., ZnS, SiO_2_ and Al_2_O_3_, or doping with metal ions can passivate surface defects and increase the PLQY of QDs. Moreover, the luminescence range and Stokes shift of QDs are effectively improved [24,25]. This paper systematically reviews the research progress of I-III-VI type QDs and their performance regulation approach. We also introduce the preparation methods and improvement strategies of multiple QDs from recent years. The research progress in solar cells, WLEDs and electroluminescent devices is also summarized, and the problems and future development are also discussed.

## 2. Properties of Multiple Quantum Dots

### 2.1. Crystal Structure

**Figure 1:**
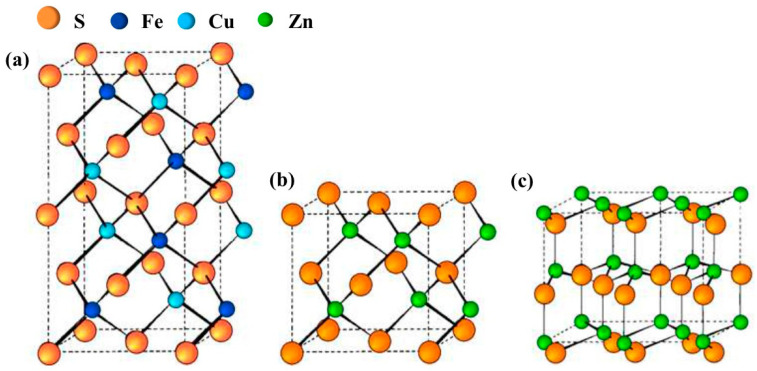
Unit cell of the (**a**) tetragonal chalcopyrite structure, (**b**) zinc blende structure and (**c**) hexagonal wurtzite structure [28].

I-III-VI QDs are composed of group I (Cu, Ag, etc.) elements, group III (Ga, In, Tl, etc.) elements and group VI (S, Se, Te, etc.) elements. They always show a tetragonal chalcopyrite structure at room temperature, while having a zinc blende structure or hexagonal wurtzite structure at high temperatures [26,27]. As shown in Figure 1a [28], the tetragonal chalcopyrite structure can be seen as the evolution of a sphalerite structure; Zn^2+^ is replaced orderly by a high valent cation (such as In^3+^, Fe^3+^, Ga^3+^, etc.) and a low valent cation (e.g., Cu^+^, Ag^+^). The decrease in structural symmetry leads to the increase of the number of atoms in the original cell from two (zinc blende) to eight (chalcopyrite), resulting in the formation of the square Bravais lattice ($I\bar{4}2d$). As shown in Figure 1b, if the Cu^+^ and In^3+^ cations are randomly distributed, the zinc blende structure is formed. In addition, when the cations are randomly distributed in the cationic sublattice, it causes the formation of a hexagonal wurtzite structure (Figure 1c) [28]. Efficient control of the reaction conditions and rational selection of organic ligands can regulate the crystal structure of I-III-VI QDs [29,30,31,32]. Omata et al. [33] pointed out that the crystal structure of CuInS_2_ QDs largely depends on the growth rate of the crystal and the bond strength between the metal monomer and the ligand molecule, and the space and size of the ligand molecule both affect the growth rate of QDs. Therefore, choosing the appropriate ligand is beneficial to controlling the crystal structure. Yang et al. [34] prepared CuInS_2_ QDs using Cu(dedc)_2_ and In(dedc)_3_ as precursors, oleylamine as an activator and oleic acid as a ligand. When dodecanethiol is employed as a ligand, the CuInS_2_ QDs show a wurtzite structure. Moreover, the stoichiometric ratio also has an equally important influence on the crystal structure of I-III-VI type QDs. Xiang et al. [35] demonstrated that the crystal structure of Cu-In-Zn-S QDs changed from a wurtzite structure to a chalcopyrite structure with a decreasing Cu/Zn ratio from 1:1 to 1:15. When the Cu/Zn ratio further decreased to 1:20, the QDs showed a zinc blende structure. The above research shows that the crystal structure of I-III-VI type QDs is affected by the synthesis method, ligands, reaction temperature, precursor, etc. Therefore, the corresponding crystal structure can be obtained by changing the experimental parameters and thus regulating the photoelectric performance.

### 2.2. Optical Properties and Luminescence Mechanism

Compared to traditional II-VI and III-V binary QDs, I-III-VI type QDs have the following three significant optical characteristics: a longer fluorescence lifetime (100~300 ns), a larger Stokes shift (300~500 meV) and a wider half-height width (>300 meV) [36,37]. Among these, the longer fluorescence lifetime is related to the larger Stokes shift and the recombination of the internal defect energy level, while the larger half-height width is mainly attributed to the component or size polydispersity of the multiple QDs [38]. For example, Yang et al. [39] believed that CuInS_2_ QDs have a large luminous linewidth, which is mainly related to the distribution of donor–acceptor distance and the interaction of photons and phonons in DAP recombination. They separated CuInS_2_ QDs with different particle size ranges through size-selective precipitation to reduce their size distribution, and found that the half-height width of the QDs was still up to 250 meV. However, Klimov et al. [40] found that the half-height width of single size distribution QDs was significantly reduced from 300 meV to 60 meV compared with the whole size distribution QDs by studying the “flicker” behavior of CuInS_2_/ZnS QDs. Therefore, they believed that the existence of randomly distributed emission centers inside the QDs was the main reason for their large half-height width. In addition, the fluorescence performance of I-III-VI QDs can be adjusted by size and composition at the same time, so that the emission spectrum can be continuously tuned within the whole visible light region [41]. Han et al. [42] controlled the size of QDs by adjusting the reaction temperature. Therefore, the emission peak of the prepared QDs was continuously adjustable at 520~700 nm. Wang et al. [43] obtained CuInS_2_ QDs with luminescent peaks covering 543~700 nm by changing the Cu/In molar ratio.

**Figure 2:**
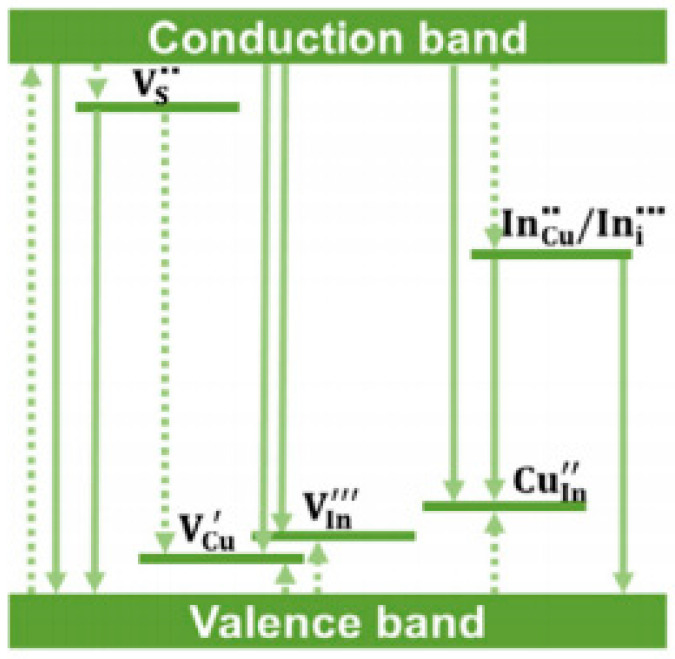
Emission mechanism schematic of CuInS_2_ QDs [47].

Due to the high mobility of group I cations and the low enthalpy of formation of V_Cu_ and In_Cu_ defects, there are a lot of defects in the interior of I-III-VI QDs, such as inverse (In_Cu_, Cu_In_), interstitial (Cu_i_, In_i_) or vacancy (V_Cu_, V_In_, V_S_) defects, which can accommodate large non-stoichiometric quantities in the structure, resulting in a variety of different emission modes [44,45,46]. At present, donor–acceptor pair (DAP) recombination (as shown in Figure 2) is the most recognized emission method of I-III-VI QDs, such as the transition from V_S_ to V_Cu_ or from In_Cu_ to V_Cu_, in which the In_Cu_ or V_S_ acts as the donor and V_Cu_ acts as the acceptor [47]. In addition, the transition from the bottom of the conduction band to the acceptor near the valence band [15,46] and the radiation recombination mode related to the surface defect state [48,49] have also been proposed. A large number of studies have shown that type I vacancy has an important impact on the luminescent properties of I-III-VI QDs. The design of group I ion vacancy type QDs through reducing the ratio of Cu/In or Ag/In helps to improve the recombination rate of carriers, and can significantly improve the luminous intensity of QDs [41,46,50,51]. In addition, Hamanaka et al. [52] believed that there were a great number of donor–acceptor type defects in QDs with smaller particle sizes, and the luminescent properties of QDs could be improved by adjusting the particle sizes.

Due to the large specific surface area of QDs, a large number of dangling bonds on the surface of QDs can be used as non-radiative recombination centers of excitons, resulting in the decline of fluorescence properties of QDs. In order to improve the PLQY value of I-III-VI type QDs, ZnS [22,53,54] or CdS [55,56] with wide band gaps are usually used to embed the QDs to limit electrons and holes to the interior of the QDs, and thus eliminate the surface states and reduce the overlap of electron/hole wave functions, which effectively improves the radiation recombination efficiency of QDs [57]. Do et al. [58] prepared Zn-Ag-In-S/Zn-In-S/ZnS core/shell/shell QDs using a multi-step thermal injection method. By inserting a layer of Zn-In-S inner shell between the Zn-Ag-In-S core and the ZnS shell as a transition layer, the lattice mismatch between the core and shell can be effectively reduced, and the surface stress of QDs can be released at the same time. Therefore, the PLQY value of the Ag-In-S/Zn-In-S/ZnS core/shell/shell QDs is up to 87%.

In addition, doping Zn^2+^ into the I-III-VI QDs to form quaternary Zn-I-III-VI alloy QDs can also significantly reduce the concentration of cation vacancies in the lattice and eliminate non-radiative recombination centers. Meanwhile, it also can improve the PLQY value and further expand the coverage range of the QD emission peaks [59,60]. Maeda et al. [61] found that the luminous intensity of CIZS QDs reached the highest value when the doping amount of Zn^2+^ reached 20~33%. At present, there have been a large number of reports on quaternary alloy QDs, such as CIZS [62,63], AIZS [64,65], CIGaS [66], etc., which are of great significance in promoting the application of multiple QDs in biological and optoelectronic fields.

## 3. Preparation of Multiple Quantum Dots

How to balance the chemical reactivity of each reaction precursor and avoid the occurrence of the phase separation phenomenon is the main problem for the successful synthesis of pure phase multiple QDs. According to the hard–soft acid–base (HSAB) theory, the reactants can be divided into acids (accepting electrons) and bases (providing electrons). These bases can be classified as “soft” or “hard” based on their ability to accept or provide electrons. The central tenet of HSAB theory is that the combination of “hard-hard” and “soft-soft” tends to form more stable compounds [67]. HSAB theory has been widely used in the colloidal synthesis of nanocrystals, and it is an important guideline for the selection of reaction precursors and ligands. In I-III-VI type QDs, there are large differences in the chemical properties of each cation (e.g., Ag^+^ and Cu^+^ are soft acids and In^3+^ is a hard acid), while the sulfur group elements (S, Se, Te) are soft bases. Therefore, how to balance the chemical reactivity between cations and avoid the formation of binary sulfide is the key to their synthesis [67]. Therefore, two or more different types of organic ligands (e.g., mercaptan, carboxylic acid or amine) are usually introduced in the synthesis process to regulate the chemical reaction activity between different cations [41], or excessive mercaptan can be used as a ligand to reduce the reactivity of the cations to the same level [68]. At present, I-III-VI type QDs can be prepared in an organic or aqueous solvent. I-III-VI type QDs synthesized by an organic phase have the advantages of good crystallinity and a high PLQY value [69]. In contrast, I-III-VI type QDs synthesized by an aqueous phase have poor crystallinity and a low PLQY value due to the low reaction temperature, but the use of water as the reaction medium also gives the synthesis process obvious advantages such as safety, environmental friendliness and biocompatibility [14].

### 3.1. Organic Synthesis

I-III-VI type QDs with good dispersion and excellent optical properties can be obtained by the organic phase synthesis method, which is due to the fact that the organic phase synthesis can provide a higher reaction temperature than the aqueous phase synthesis, resulting in a better crystallinity of the QDs [70]. Table 1 summarizes the optical properties of I-III-VI type QDs synthesized by organic phases in recent years.

The hot injection method is one of the most commonly synthetic routes for preparing I-III-VI type QDs. Based on the principle that QDs with a good dispersion can be obtained by instantly increasing the concentration of monomers in the solution, the anion precursor is quickly injected into the high temperature reaction solution containing the cationic precursor, forcing the concentration of the reaction precursor to instantly oversaturate and exceeding the nucleation critical point, and crystal nuclei are quickly obtained. This method can separate the nucleation and growth of QDs and control the monodispersion and size of QDs well [45,78]. Shen et al. [79] synthesized AIS QDs using the hot injection method using oleylamine (OAm) and 3-mercaptopropionic acid as organic ligands. The average diameter of the AIS QDs was 5.0 ± 0.5 nm and the PLQY could reach up to 8.34%. Mohan et al. [80] synthesized AIS QDs with octaecene as an organic solvent via the hot injection method. By coating with a ZnS shell, the band gap of the QDs increased from 2.32 eV to 3.30 eV.

**Figure 3:**
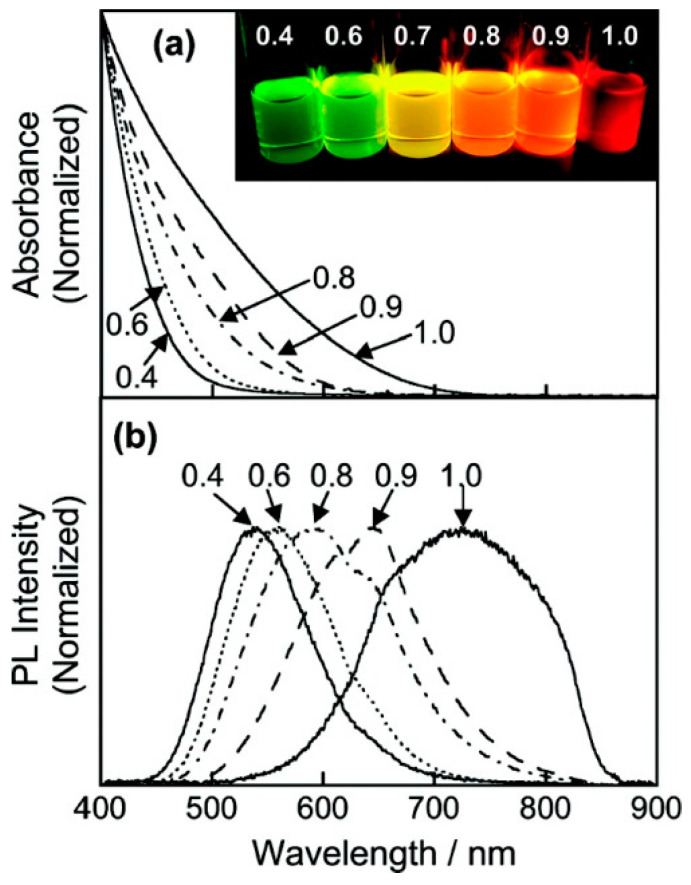
Absorption spectra (**a**) and PL spectra (**b**) of AIS/ZnS QDs obtained from the decomposition of precursor (AgIn)_x_Zn_2(1−x)_(S_2_CN(C_2_H_5_)_2_)_4_ with different x values. The inset in panel a shows photographs of UV-illuminated AIS/ZnS nanoparticle solutions. Reprinted with permission from ref. [82]. Copyright 2007, American Chemical Society.

The single-source precursor pyrolysis method is to heat the single-molecule precursor containing all the elements in the QDs at a high temperature in an organic solvent, and obtain the corresponding QDs through the decomposition of the precursor [81]. Castro et al. [15] prepared CIS QDs with a particle size of 2~4 nm by the pyrolysis of a single precursor (PPh_3_)_2_CuIn(SEt)_4_ at 200 °C using hexyl mercaptan as a ligand and octyl glycol titanate as a solvent. The PLQY of the CIS QDs could reach 4.4%. Torimoto et al. [82] prepared AIS/ZnS QDs with the pyrolysis precursor (AgIn)_x_Zn_21−x_(S_2_CN(C_2_H_5_)_2_)_4_. As shown in Figure 3, the emission wavelength of the AIS/ZnS QDs shifts from 720 nm to 540 nm by adjusting the proportion of chemical components, and the PLQY reaches up to 24%.

The heating up method (also known as the “one-pot method”) provides a new way for the controllable batch preparation of high-quality multiple QDs. This method dissolves the metal ion precursor, ligand and anionic precursor in a solvent to obtain a uniform solution, and directly heats the above mixed solution to a specific temperature for a certain time to obtain multiple QDs [83]. Liu et al. [65] used oleic acid (OA) and dodecyl mercaptan (DDT) as organic ligands to synthesize AIS QDs using the heating up method, and the PLQY reached 53.1%. AIZS QDs were prepared via doping Zn^2+^ into AIS QDs. By adjusting the doping amount of Zn^2+^, the PLQY of AIZS QDs was up to 60.3% and the fluorescence lifetime was increased from 173.5 ns to 190.5 ns. Zhang et al. [84] prepared CIZS QDs with metal ion acetates as the reaction precursors, OAm as the activator and DDT as the ligand using a one-pot method at 220 °C. The emission peaks of the CIZS QDs were continuously tuning from 450 nm to 810 nm, and the PLQY could reach 85% after ZnS coating. Hu et al. [85] prepared AIS QDs in the non-polar organic solvent octadecene using glycerol as a microwave absorber. By coating the ZnS shell, the PLQY of the AIS/ZnS QDs was further increased from 13.66% to 33.10%. A white light emitting diode (WLED) was prepared with AIS/ZnS QDs mixed with commercial phosphor and then coated on a blue LED. The color rendering index (CRI), correlation color temperature (CCT) and luminous efficiency (LE) of the device were 87.6, 3361 K and 58.83 lm/W, respectively, indicating the WLED has a good application prospect in the lighting field.

### 3.2. Aqueous Synthesis

Although organic phase preparation provides a feasible way to obtain high-quality I-III-VI type QDs, their surfaces are modified by long chain organic molecules, resulting in poor solubility of the QDs in an aqueous phase, which greatly limits their application in aqueous phase environments. Direct synthesis of hydrophilic QDs is an effective way to solve these problems. Aqueous synthesis usually requires hydrophilic sulfhydryl groups as ligands to synthesize the QDs, such as glutathione and cysteine [86,87]. Table 2 summarizes the size and optical properties of the I-III-VI type QDs obtained by aqueous phase synthesis methods in recent years.

May et al. [97] prepared Gd-doped AIS QDs using sodium citrate and thioglycolic acid as the ligands in the aqueous phase, and the emission peak of the QDs could be tuned in the range of 583~626 nm by adjusting the Gd doping amount. Xue et al. [98] prepared AIS QDs with glutathione as the ligand in the aqueous phase, which can be used to measure Pb^2+^ concentration in water. In the range of 9~530 nM, there was a good linear relationship between the Pb^2+^ concentration and the relative fluorescence quenching of the AIS QDs. The detection limit was 4 nM and the recoveries were 92.2~102.6%. Stroyuk et al. [99] prepared AIS QDs in the aqueous phase with glutathione as the ligand, and the particle size of the AIS QDs was 2–3 nm. By adjusting the width of the band gap, the PLQY of the AIS QDs could reach 32%. Liu et al. [100] synthesized CIZS QDs using a microwave assisted-hydrothermal method and mercaptopropionic acid as the ligand. The PLQY of the CIZS QDs reached 23% after being coated with ZnS, and they were successfully used for Hela cancer cell imaging.

## 4. PLQY Improvement Strategies of I-III-VI Type QDs

### 4.1. Doping of I-III-VI Type QDs

Multiple QDs are widely used in catalysis, charge storage and thermoelectric conversion because their emission peak range and intensity can be tuned by changing their size and cationic composition [101,102]. However, due to the different ionic activities of multiple QDs, the crystal structure is more prone to defects, resulting in a lower PLQY of multiple QDs than the traditional binary QDs. This can be improved by doping transition metal ions or rare metal ions (e.g., Mn^2+^, Cu^+^, Co^2+^, Ni^2+^, Ag^+^, Au^3+^, etc.). The doped QDs not only retain optical properties, but also have the advantages of a high PL intensity, broad luminescence range and large Stokes shift. However, the doping mechanism is complex and certain doping structures are difficult to obtain. For example, Mn^2+^ can be doped into CdS and ZnSe QDs, but it is difficult to dope into CdSe QDs [103,104,105,106]. In view of this phenomenon, Erwin et al. [107] attributed it to the “self-purification” theory, that is, impurities will be discharged by QDs. Meanwhile, doping efficiency is determined by three main factors: surface morphology, nanocrystal shape and surfactant.

#### 4.1.1. Doping Mechanism

**Figure 4:**
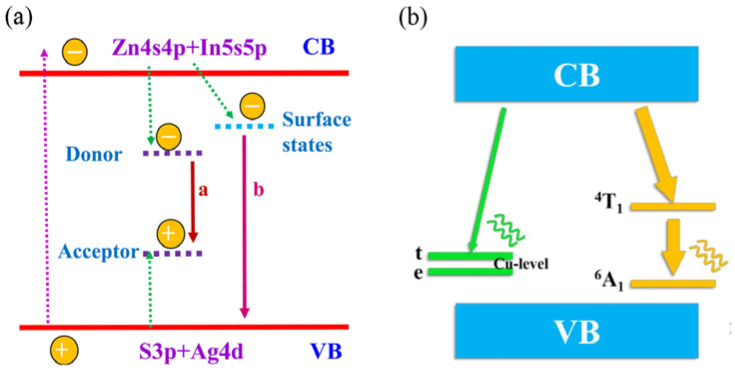
Emission mechanism of (**a**) AIZS QDs [108], and (**b**) Mn^2+^ and Cu^+^ doped ZnInS QDs [109].

At present, Zn^2+^, Mn^2+^ and Cu^+^ are the main transition element ions used for doping I-III-VI type QDs. The luminescence mechanism of AIZS QDs obtained by doping AIS with Zn^2+^ is shown in Figure 4a [108]. The valence band (VB) of AIZS QDs is composed of hybrid orbitals of S 3p and Ag 4d, and the conduction band (CB) is composed of hybrid orbitals of Zn 4s4p and In 5s5p. The Ag-S and In-S bonds are easily broken, and Zn^2+^ is prone to cation exchange with Ag^+^ and In^3+^ in QDs. Therefore, changing the doping amount of Zn^2+^ can widen the band gap of QDs and change its PL properties. In Figure 4a, path a is the compound process of the intrinsic trap state (donor–acceptor pair, DAP), and path b is the capture process of photogenerated electrons by surface defects. Most electrons and holes of the AIZS QDs are recombined in the way of path a, and the radiation recombination efficiency can be improved by reducing surface defects [13,79]. The luminescence mechanism of Mn^2+^ and Cu^+^ doping is shown in Figure 4b [109]. After doping Mn^2+^ or Cu^+^, the photoexcited electrons in the VB do not fall back to the VB directly, but first transfer energy to the ^4^T_1_ state of Mn^2+^ (or the ^2^T_2_ state of Cu^+^), and then fall back to the ^6^A_1_ state from the ^4^T_1_ state, at the same time the photon is released. Since the energy released is less than the bandgap energy of the main QDs, the Stokes displacement increases and the self-absorption phenomenon is greatly reduced. It can also be seen from Figure 4b that the d-d transition emission of Mn^2+^ does not change the band gap of the main QDs, and is independent of the shape, size and properties of the main QDs, so the stability of the QDs is greatly increased after doping Mn^2+^ [110]. Compared to Mn^2+^-doped QDs, Cu^+^-doped QDs have a larger Stokes shift, and the emission of Cu^+^-doped QDs is closely related to the band gap energy. The main band gap, size and composition of QDs can be changed and the shape can be controlled based on the quantum confinement effect. Therefore, the emitted light color can be further extended from the visible light wavelength range to the near-infrared light wavelength range [110].

#### 4.1.2. Zn^2+^-Doped I-III-VI Type QDs

**Figure 5:**
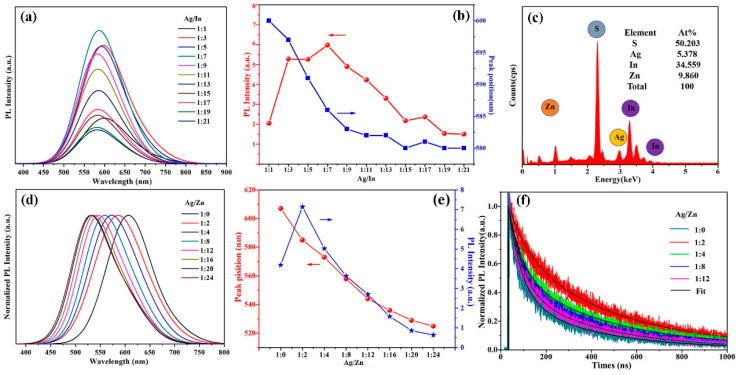
(**a**) PL emission spectra, (**b**) evolution of the PL intensity and peaks position, (**c**) EDS spectrum, (**d**) normalized PL emission spectra, (**e**) evolution of the PL intensity and peaks positions and (**f**) PL decay profiles of the AIZS QDs with different Ag/Zn ratios [116].

CIS and AIS QDs are the most important members of the I-III-VI type QDs. As a direct band gap semiconductor material, CIS QDs have a band gap of 1.5 eV, which is in line with the optimal absorption range of sunlight and has a large absorption coefficient [45]. The direct band gaps of AIS QDs range from 1.54 to 2.03 eV and vary depending on whether the crystal structure is orthogonal or square [13]. Doping Zn^2+^ in CIS or AIS QDs widens the band gap, and thus tunes the emission peak range in the visible to near-infrared range. For example, Zhang et al. [111] directly synthesized CIZS QDs with different sizes and non-stoichiometric sizes using copper acetate, zinc acetate, indium acetate and sulfur powder as the raw materials. The emission peak can be tuned from the visible region to the near-infrared region, and the PLQY exceeds 70% without further surface modification. Chi et al. [112] compared the optical properties of CIS and CIZS QDs, and found that the absorption spectra and emission spectra of QDs were blue-shifted and the PL intensity was improved when the doping amount of Zn^2+^ was 10%. Song et al. [113] synthesized AIZS QDs in aqueous solution using a cation exchange method. By adjusting the doping amount of Zn^2+^, the PL emission could be shifted from 483 nm to 675 nm and the PLQY reached 44%. Mansur et al. [114] used carboxymethyl cellulose (CMC) as a ligand to prepare AIS and AIZS QDs, and the PLQY values were 1% and 4%, respectively. The authors’ research group prepared CIZS QDs in an aqueous phase using a reflux method and an ionic liquid-assisted microwave method, respectively. The emission wavelength can be continuously adjusted in the range of 588~668 nm and 579~677 nm by changing the proportion of metal ions, respectively [62,115]. Meanwhile, we also prepared AIZS QDs using an ionic liquid-assisted hydrothermal synthesis method, as shown in Figure 5. The luminescence peak of the AIZS QDs was varied from 600 to 580 nm when the Ag/In molar ratio was changed from 1/1 to 1/21. A clear blue-shift of the PL peak from 608 nm to 525 nm was observed by decreasing the Ag/Zn molar ratio from 1/0 to 1/24, indicating that the increase of the Zn^2+^ content caused the increase of the band gap. Correspondingly, the average fluorescence lifetimes decreased from 454.43 ns to 359.67 ns with the decrease of the Ag/Zn ratios from 1:2 to 1:12, indicating that a proper Ag/Zn molar ratio was beneficial to improving the radiative recombination of QDs [116].

In the preparation of Zn^2+^ doped I-III-VI type QDs in a liquid phase, the temperature is one of the main factors affecting the diffusion of Zn^2+^. Tang et al. [117] found that the higher the crystallinity of AIS QDs, the more difficult the diffusion of Zn^2+^ into AIS QDs. Therefore, the diffusion of Zn^2+^ can be tuned by changing the crystallinity of AIS, so as to adjust the band gap of AIS nanocrystals. This group also controlled the content of Zn^2+^ in the AIS QDs by changing the preparation temperature. When the temperature increased from 120 °C to 210 °C, the emission wavelength shifted from 680 nm to 520 nm, and the PLQY also enhanced from 32% to 41% [118].

In addition to the reaction temperature, the reaction time is also one of the most important parameters affecting the diffusion of Zn^2+^ and thus changing the luminescence properties. Wang et al. [119] injected Zn^2+^ into ternary AIS QDs using a one-pot method, and the emission peak shifted from 638 nm to 602 nm within 0.5 min, and then to 584 nm after a 15 min reaction. The highest PLQY of the QDs obtained could reach about 70%. Kong et al. [120] synthesized CIZS QDs in the non-polar solvent octadecene via a two-step method. The size of the CIZS QDs could be regulated by changing the growth time of the QDs, and the hybrid emission of QD/PSF was realized by spinning the CIZS QDs onto plasma silver films (PSFs). This method improves the excitation rate or emissivity of the QDs and increases the PL peak intensity by 45 times.

#### 4.1.3. Mn^2+^-Doped I-III-VI Type QDs

Doping transition metal ions into QDs usually introduces the related energy levels of dopants in the band gap of crystals, causing changes in the optical properties of doped QDs [121,122]. When QDs absorb photons to produce electron-hole pairs, the electrons and holes form bunched states (excitons) due to coulomb gravity. Excitons transfer energy to the doping level, causing electron-hole recombination and emitting fluorescence [123]. There is another case where only electrons or holes are transferred to the dopant site, resulting in a charge transfer and electron-hole recombination, and are therefore still subject to the dopant [124].

The optical properties of doped QDs are determined by the interaction between the wave function of quantum bound excitons and the doping center [125]. Therefore, the concentration of dopants in QDs has an important effect on controlling the properties of doped nanocrystals [126]. Abate et al. [5] synthesized Mn^2+^-doped CISe (Mn:CISe) QDs using the microwave method in aqueous solvent. Compared to the undoped QDs, Mn^2+^-doped QDs have a wider absorption range and stronger visible light absorption. Although the position of the emission peak does not change significantly, the emission intensity decreases. Hua et al. [127] also noted that when Mn^2+^ was doped into CIS QDs, the PL intensity of the QDs gradually decreased with the increase of Mn^2+^ content. Because Mn^2+^ doping promoted the non-radiative recombination process [128], and the excitation energy was transferred from an Mn^2+^ to the nearest Mn^2+^ through non-radiative transition and several migration steps, it finally reached the quenched position (e.g., defect state) [129]. In addition, Kim et al. [130] made the emission light of doped Zn-Cu-Ga-S QDs change from blue to red–white by adjusting the concentration of Mn^2+^.

In the case of Mn^2+^ doping, the radiation process originates from the transfer of exciton energy from the subject QDs to the local Mn^2+^, followed by the electron transition of the d orbital (^4^T_1_-^6^A_1_) of Mn^2+^. Therefore, the excitation energy of Mn^2+^-doped QDs becomes relatively insensitive to the size and composition of the QDs [131]. Doped multiple QDs do not necessarily produce a single PL associated with the dopant, because the radiative recombination of undoped QDs involves an intrinsic defect site as an acceptor and/or donor. The emission of doped I-III-VI type QDs is captured or transferred to intrinsic and external lattice defects (i.e., dopants) by means of a recombination of carrier or excitation energy competition, resulting in an overlap of the main defect emission and dopants [130].

#### 4.1.4. Cu^+^-Doped I-III-VI Type QDs

For Cu^+^-doped QDs, the emission is related to the transition from the CB of QDs to the d energy level of Cu, that is, the recombination of the electron in CB doped with copper QDs with the hole in Cu ^2^T_2_ state leads to exciton relaxation, so the fluorescence emission of the Cu^+^-doped QDs is related to band gap energy level splitting, thus affecting the optical properties of QDs [132].

**Figure 6:**
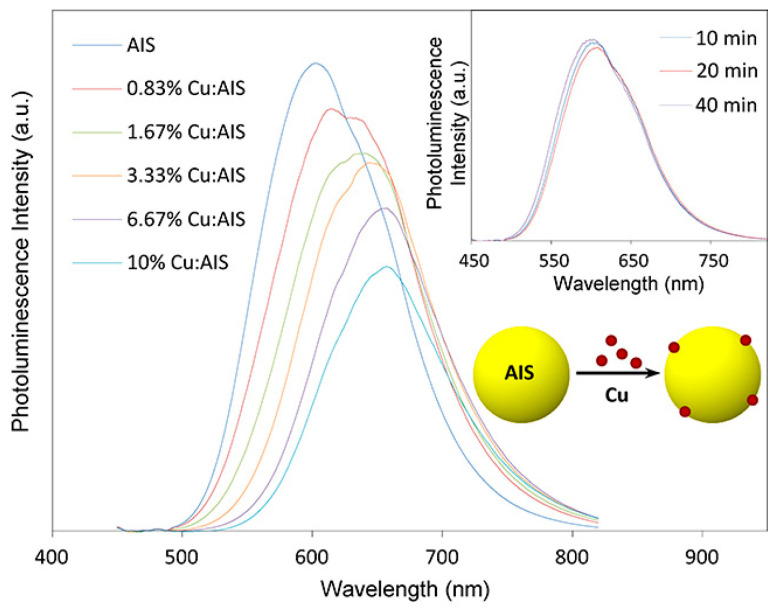
Effect of surface-doped Cu concentration on the PL spectra of Cu:AIS (Cu:AIS) QDs. The inset data plot shows that the PL spectra of undoped AIS QDs evolve insignificantly during the growth time [133].

Chen et al. [133] doped Cu^+^ into AIS QDs (Cu:AIS) to study the influence of the Cu^+^ doping concentration on the PL spectra of QDs, as shown in Figure 6. Compared with pristine AIS QDs, the PL peak wavelength of 6.67% Cu^+^-doped AIS QDs showed a continuous red shift at 600~660 nm and the PLQY decreased from 28.9% to 19.7%. With a further increase of Cu^+^ doping concentration, the PL peak red shift was not obvious and the PLQY decreased further. Meanwhile, Cu^+^ doping could prolong the PL lifetime of AIS QDs. Raevskaya et al. [134] observed a similar phenomenon in Cu:AIS QDs prepared in an aqueous solution. The PL peak of the QDs continued to red-shift and the intensity decreased rapidly with Cu^+^ doping into AIS QDs. The PL intensity of Cu:AIS QDs reaches the highest value when Cu:Ag:In:S = 1:1:7:10, and the center of the emission peak is about 780 nm (1.6 eV). On the other hand, the PLQY value of Cu:AIS QDs decreases with the increase of Cu^+^ doping concentration, because more defects are generated in the Cu:AIS lattice, and the high concentration makes Cu-Cu interactions stronger, leading to fluorescence quenching [135].

Guchhait et al. [136] systematically studied the influence of Cu^+^ doping on the absorbance and crystallinity of Cu:AIS QDs. With the increase of Cu^+^ doping, the absorption peak of QDs continuously red-shifted from 670 to 850 nm, which is due to the interaction between the 3d state of Cu and the VB edge of the main material. In addition, Cu^+^ can fill the vacancy inherent in the AIS lattice and partially replace Ag^+^, thus improving the crystallinity of AIS QDs.

#### 4.1.5. Co-Doped I-III-VI Type QDs

In addition to single metal ion doping multiple QDs, multi-ion co-doping can also improve the optical properties of multiple QDs. Zhou et al. [137] prepared CIS QDs co-doped with Zn^2+^ and Mn^2+^. The excitation peak slightly red-shifted and the full width at half maximum (FWHM) decreased gradually with the increase of reaction time. All the doped samples fluoresced red light, and the fluorescence intensity was higher than that of the undoped samples. Galiyeva et al. [138] prepared AIS QDs co-doped with Zn^2+^ and Mn^2+^ using a one-step method. The PL luminescence peak of the QDs red-shifted with the increase of the Mn^2+^ concentration, which may be due to an Mn^2+^-related trap state. Co-doped AIS QDs also exhibit ferromagnetic and paramagnetic properties, as well as long PL lifetimes, which enable them to distinguish signals from rapidly decaying background PL and biological system PL, and have great potential for development in bioimaging applications.

**Figure 7:**
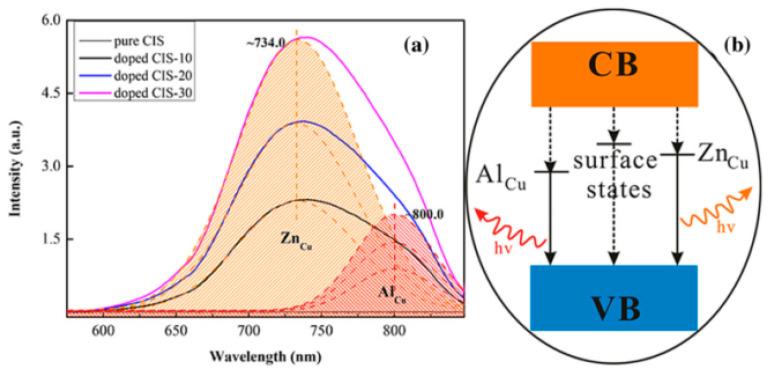
(**a**) PL spectra of pure and doped CIS QDs including doped CIS-10, doped CIS-20 and doped CIS-30 under 460 nm excitation, and Gaussian deconvolutions of PL peaks from the three doped CIS QDs. (**b**) The model of the proposed electronic transition responsible for PL emission of pure and doped CIS QDs [139].

Zhu et al. [139] synthesized CIS QDs co-doped with Zn^2+^ and Al^3+^ using the cation exchange method. Ion doping significantly improved the PL performance of CIS QDs, as shown in Figure 7. As the doping reaction time increased from 10 to 30 min, the intensity of the luminescence peaks increased significantly, and two luminescence peaks (734 nm and 800 nm) were observed, corresponding to Zn_Cu_ and Al_Cu_ defects, respectively. Meanwhile, the proportion of peaks at 734 nm increased continuously. This is because the Zn^2+^ and Al^3+^ dopants on the surface can bind with S atoms and reduce the number of suspended bonds, thus inhibiting non-radiative recombination. The doping rate of Zn^2+^ increases with the extension of doping time, leading to the increase in the proportion of the luminescence peak at 734 nm.

The PL properties of co-doped QDs depend on the interaction between the two dopants. For example, for Cu^+^ and Mn^2+^ co-doped ZIS QDs, less Mn^2+^ is conducive to the energy transfer of the excited energy level of Mn^2+^ to the luminescent center of Cu^+^, generating additional excitons and thus increasing the PL intensity [109]. With the increase of the Cu^+^ doping concentration, the emission peak of Mn^2+^ and the PL intensity decrease, indicating that the energy difference between the Mn ^4^T_1_ and ^6^A_1_ states decreases. This mechanism of dopant interaction can be widely applied to other co-doped QDs [109,140].

### 4.2. Surface Coating of I-III-VI Type QDs

Due to the small particle size of QDs, a larger number of surface-suspended bonds lead to the fluorescence quenching of QDs [141]. It is usually necessary to modify the surface of QDs with various organic ligands to passivate the surface defects. However, the organic ligands cannot simultaneously passivate the surface electrons and hole trap states of QDs. Meanwhile, they are susceptible to water, oxygen erosion and photodegradation, so the long-term effective luminescence and stability of QDs cannot be ensured. Thirdly, due to the “adsorption-desorption” equilibrium between the ligands and QDs, and steric effects between the ligands, there are still many defects on the surfaces of QDs, resulting in the low PLQY of QDs [141]. The ideal way to solve the above problems is to grow an inorganic shell material on the surface of QDs to form core–shell structure QDs, which can effectively passivate the surface state of QDs and thus improve the fluorescence performance of QDs [142].

Due to the wide band gap (~3.7 eV) of the ZnS shell and its low lattice mismatch (2.2%) with the I-III-VI type QDs, ZnS is used as a shell material to eliminate the defects of QDs and passivate their surface, resulting in an improvement of fluorescence properties. Jain et al. [22] synthesized CIS QDs using 3-mercaptopropionic acid (MPA) as the ligand using a hydrothermal method. The particle diameter was 6.6 nm. After the CIS QDs were coated with ZnS, the average size of the CIS/ZnS QDs increased to 11.2 nm and the PLQY increased from 6.95% to 17.19%. Bora et al. [143] synthesized CIZS QDs and CIZS/ZnS QDs in a mixed solvent of oleylamine and octylamine using a one-pot method with DDT as the ligand. The growth of the ZnS shell led to a further increase of PLQY from 9% of the CZIS QDs to 29% of the CZIS/ZnS QDs. Meanwhile, the emission peak of CZIS/ZnS QDs can be adjusted in the range of 650~710 nm by changing the composition of the QDs. Vitshima et al. [144] synthesized AIS QDs using a hydrothermal method with glutathione as the ligand, and the PLQY of the AIS QDs was 31.6%. The luminescence peak of the ZnS-passivated QDs shifts continuously from 582 nm to 525 nm, while the PLQY of the AIS/ZnS core/shell QDs is 35.4%. Soheyli et al. [145] used N-acetyl-l-cysteine as the capping agent to synthesize Ag-doped ZCdS/ZnS core–shell QDs with a one-pot method. At a molar ratio of Zn:Cd between 2:0 and 0:2, the PLQY of the Ag:ZCdS/ZnS core–shell QDs could be adjusted in the range of 5~39%. Shim et al. [146] synthesized CIS, ZCIS and ZCIS/ZnS QDs with a continuous hot injection method, and their PLQYs were 2%, 30% and 70%, respectively.

ZnSe is another commonly used shell material in the fabrication of core–shell structure QDs. Oluwafemi et al. [147] synthesized water-soluble AISe QDs and AISe/ZnSe QDs via an electric pressure pot method using thioglycolic acid (TGA) and gelatine as the ligands. The average particle sizes were 2.8 nm and 3.2 nm, respectively. Meanwhile, the PLQY of the QDs increased from 0.62% to 5.6%. The enhancement of PLQY for the core–shell structure QDs is mainly due to the evolution of electronic structure and the suppression of defect states, which enhances the radiation channel and reduces the non-radiation channel of QDs [146].

## 5. Application of I-III-VI Type QDs

### 5.1. Solar Cells

**Figure 8:**
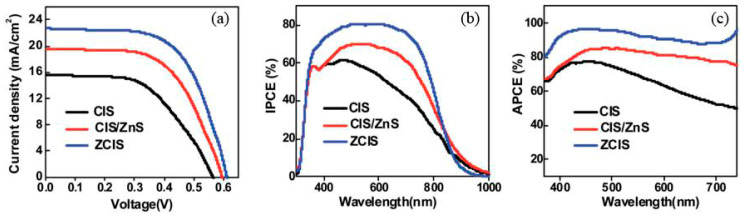
(**a**) J-V curves of optimal solar cells based on CIS, CIS/ZnS and ZCIS photosensitizers; (**b**) IPCE and (**c**) APCE. Reproduced with permission from ref. [152]. Copyright 2018, The Royal Society of Chemistry.

The excessive use of fossil energy causes global energy exhaustion and environmental pollution. It is urgently necessary to develop renewable and clean energy. Among them, quantum sensitized solar cells (QDSSCs) have attracted attention for their outstanding advantages such as high conversion rate and low cost, and their theoretical power conversion efficiency (PCE) is as high as 44%, making them promising candidates for the next generation of solar cells [148]. The band gap width of I-III-VI type QDs (AIS, CIS, CISe, etc.) can be adjusted by changing their components, which overcomes the disadvantage of binary QDs only relying on size adjustment. Therefore, they greatly increase the adjustable range of optical properties, and are an ideal substitute material for highly toxic cadmium-based QDs. Cheng et al. [149] synthesized AIS QDs using the hot injection method. The PLQY of the AIS QDs was about 7~10%, and they were connected to a mesopore TiO_2_ surface by dual-functional linking molecules. Due to the excessive charge recombination of the AIS QDs, the assembled solar cells had a short circuit current of 0.49 mA/cm^2^ and an open circuit voltage of 0.24 V, and the PCE was only 0.05%. In addition, the long and heavy hydrocarbon chain covering AIS QDs prevents its maximum coverage on the TiO_2_ surface, resulting in a low conversion performance of devices. Based on this, Kadlag et al. [150] synthesized ligand-free AIS QDs using formamide as an organic solvent, and the open circuit voltage and PCE were 0.45 V and 0.8%, respectively. Wang et al. [151] synthesized AIS/In_2_S_3_/ZnS in the aqueous phase. The In_2_S_3_ and ZnS inhibited charge recombination, and the assembled solar cells had a PCE of 0.7%. Zhang et al. [60] synthesized AIS QDs using oleamine as a surfactant using a simple and green method. The PCE of the assembled solar cells was 2.46%. The introduction of Zn^2+^ into AIS QDs can optimize the defect state and inhibit charge recombination, and the PCE of solar cells assembled by AIZS QDs reaches 4.50%. Yue et al. [152] compared the PCE of solar cells assembled with CIZS, CIS/ZnS and CIS QDs as photosensitized materials, as shown in Figure 8. The PCE of QDSSCs using CIZS QDs as the photosensitizer was 8.47%. Compared with CIS/ZnS and CIS-based QDSSCs, the PCE of the CIZS-based QDSSCs increased by 21% and 82%, respectively, under AM 1.5 G simulated sunlight irradiation. The core–shell structure has a certain inhibition effect on charge recombination and a shell with a wide band gap will also slow down the charge extraction rate, resulting in the deterioration of photovoltaic performance [153,154]. In contrast, alloying structures is conducive to inhibiting charge recombination loss and accelerating electron injection of QDs into TiO_2_ electron acceptors due to uniform electronic structures [154,155].

Compared to AIS and CIS QDs, CISe QDs have a larger exciton Bohr radius (10.6 nm) and a narrower band gap (1.04~1.5 eV), which can extend the light absorption range to the near-infrared (NIR) region, so it has widely attracted the attention of researchers in recent years [156]. Du et al. [157] synthesized high quality Cu-In-Zn-Se (CIZSe) QDs, and the average PCE of QDSSCs based on these QDs was 11.66%. Zhang et al. [158] synthesized CIZSe QDs with high quality and controlled components. The PCE of the prepared QDSSCs could reach 12.57% under the irradiation of 1.5 G simulated sunlight with a Cu/In molar ratio of 0.7. Zhang et al. [159] synthesized CIZSe QDs using ascorbic acid (AA) as the ligand. The addition of AA increases the band gap and thus the charge recombination process is inhibited. The efficiency of CIZSe cells with copper and titanium electrodes reached 10.44% and 13.85%, respectively.

In addition, narrowing the band gap of QD sensitizers can expand the range of light acquisition, thus improving the solar energy utilization rate of QDSSCs. In order to improve light collection without sacrificing electron injection, a common strategy has been proposed to combine two or more QDs to develop co-sensitized photoanodes, such as CIS/CdS [160]. Selecting appropriate QDs with complementary photoelectric properties as co-sensitizer and optimizing the assembly of the QDs and TiO_2_ thin films are expected to achieve a breakthrough in PCE. Wang et al. [161] deposited CIZSe and CdSe QDs on mesopore TiO_2_ films. Due to the synergistic effect of CIZSe with a wide light absorption range and CdSe QDs with a high extinction coefficient, the co-sensitized photoanode showed an optimal performance as the PCE reached 12.75% under standard AM 1.5 G sunlight irradiation.

In the past decade, the PCE of QDSSCs has reached about 12% through the design and adoption of new QD sensitizers, inhibition of charge recombination, improvement of QD loading capacity and other measures [162]. However, it is still urgently needed to improve the PCE of QDSSCs compared with perovskite solar cells and other emerging solar cells. Exploring high-quality QDs sensitizers, appropriate alloying strategies and adjusting the balance among band structure, composition and defects are necessary conditions for further development of QDSSCs.

### 5.2. White Light-Emitting Diodes

Since the implementation of the Kyoto Protocol in 1997, energy consumption and greenhouse gas emissions have become major global challenges, and the public, government, industry and academia have paid great attention to energy-saving technologies. Lighting is the second largest energy source used in buildings. In the United States, lighting accounts for 22% of electricity use and 8% of all energy use [163]. In the UK, different types of lighting consumed around 8 TWh of energy in 2018 [163]. It is well known that traditional lighting devices lead to energy waste due to low power conversion efficiency. WLEDs, as solid-state lighting devices, have attracted wide attention for their advantages of high efficiency, environmental protection and energy saving [164]. At present, the common white light emission is realized by coating yellow phosphor on the blue LED. However, due to the lack of red light emission, the color rendering index of the WLEDs device is relatively low [165]. At the same time, the traditional phosphor particles are too large to cause the self-scattering effect. Therefore, it is necessary to develop new fluorescent materials to further improve the performance of WLEDs [166].

QDs are ideal light cover layer materials for the fabrication of WLEDs due to their long fluorescence life, high PLQY, large Stokes shift and wide excitation spectrum [167]. Ruan et al. [168] synthesized green and red emission AIS/ZnS QDs via the hot injection method. The QDs were mixed and coated on blue GaN chips to fabricate the WLED devices. As the current increases from 50 mA to 400 mA, the electroluminescence (EL) spectrum and FWHM of the device have few changes, indicating that AIS/ZnS QD-based WLEDs are fairly stable under typical LED operating currents. Su et al. [169] synthesized AIS QDs via a microwave-assisted aqueous phase method using glutathione as the ligand. After coating with a ZnS shell, the PLQY of the AIS/ZnS QDs increased from 29% to 58.27%, and the QDs could emit green, yellow and red light by adjusting the Ag/In molar ratios. The AIS/ZnS QDs were embedded in polyacrylamide hydrogel, to achieve a flexible luminous film, and combined with a blue InGaN chip to fabricate WLEDs. The WLEDs showed a CRI of 87.5 and CCT of 3669 K, and the CIE coordinate was (0.39, 0.36) under a 20 mA operating current. Kang et al. [170] synthesized water-soluble AIS/ZnS QDs in an electric pressure cooker using gelatin as the coating agent, and then assembled them into WLED devices through a simple drip drying process. When the working current was 20 mA, the WLED showed an LE of 39.85 lm/W, CCT of 2634 K and CRI of 71.

**Figure 9:**
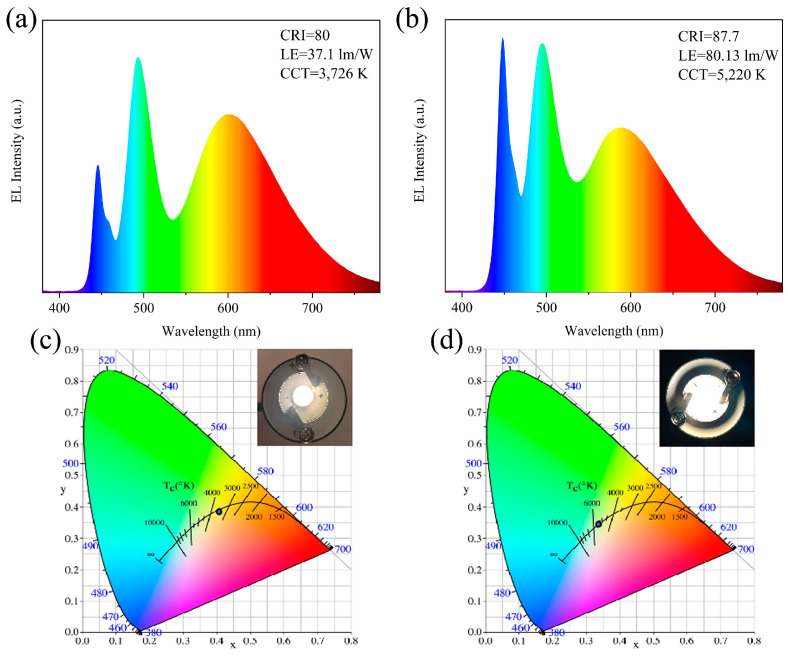
EL spectra and CIE coordinates of (**a**,**c**) AIS QD and (**b**,**d**) AIS/ZnS QD-based WLED (inset photos are QD-based WLEDs lighting at 20 mA) [14].

The authors’ research group synthesized AIZS QDs using an ionic liquid-assisted hydrothermal method, and the LE of the related WLED devices reached 85.2 lm/W [116]. In addition, CIZS/ZnS/PVP composite QDs were prepared with the ionic liquid-assisted microwave method. The surface suspension bond of the QDs was etched by F^−^, released by the ionic liquid during the reaction. The surface defect state of the QDs was passivated sufficiently by ZnS, and the PLQY increased from 6.2% to 31.2%. The LE of the related WLED devices was as high as 90.11 lm/W, and CRI and CCT were 87.2 and 4977 K [171], respectively. Meanwhile, the AIS QDs and AIS/ZnS QDs were synthesized with the microwave-assisted hydrothermal method using ionic liquid as the absorbent, for which the PLQYs were 27.67% and 35.66%, respectively. As shown in Figure 9, the AIS QD-based WLED showed a CRI of 80, CCT of 3726 K and LE of 37.1 lm/W, while the AIS/ZnS QD-based WLED had a CRI of 87.7, CCT of 5220 K and LE of 80.13 lm/W (2.16 times higher than the AIS QD-based WLED) [14].

### 5.3. Electroluminescence Devices

The QDs have the characteristics of good tunability, high luminous efficiency and strong optical stability. Therefore, they can be integrated into electroluminescent devices after simple solution processing, i.e., spin coating or inkjet printing. The luminescence mechanism of EL devices is usually explained by an injection luminescence model, i.e., holes and electrons are injected into the semiconductor device through the anode and cathode, respectively, and they are combined to emit light in the luminescent layer through the carrier transport layer [172,173]. In 1994, Alivisatos et al. [174] proposed an ITO/PPV/CdSe/Mg-structure EL device. Due to unbalanced carrier injection in the device, its external quantum efficiency (EQE) was lower than 0.01%. Subsequently, Peng et al. [175] solved the problem of imbalance between positive and negative carrier injection by inserting a PMMA insulation layer between the luminous layer and ZnO layer. The EQE was up to 20.5%, which could work continuously for more than 100,000 h at 100 mA/cm^2^. Chen et al. [176] inserted a ZnMgO/Al/HATCN/MoO_3_ internal connection layer into the device to effectively improve the generation and injection ability of carriers in the functional layer, and successfully prepared high-performance red, green and blue EL devices with the EQE upped to 23.1%, 27.6% and 21.4%, respectively.

However, the Cd-based QDs used in the luminescent layer contain heavy metal elements, which seriously restrict their commercial application. The European Union has banned such elements in industrial products. Therefore, the new green I-III-VI type QDs have widely attracted the attention of researchers in recent years. Multiple I-III-VI QDs have gained immense interest among researchers as less toxic and environmentally friendly elements have been used as compared to binary II-VI QDs. Yang et al. [177] prepared EL devices with the structure of ITO/PEDOT:PSS/PVK/QDs/ZnO/Al using CIS/ZnS QDs as a luminous layer, PEDOT:PSS as a hole injection layer, PVK as a hole transport layer and ZnO as an electron transport layer. By adjusting the thickness of the QD luminous layer, the device had a maximum brightness of 1564 cd/m^2^, current efficiency of 2.52 cd/A, EQE of 1.1% and an operating voltage of 5 V.

**Figure 10:**
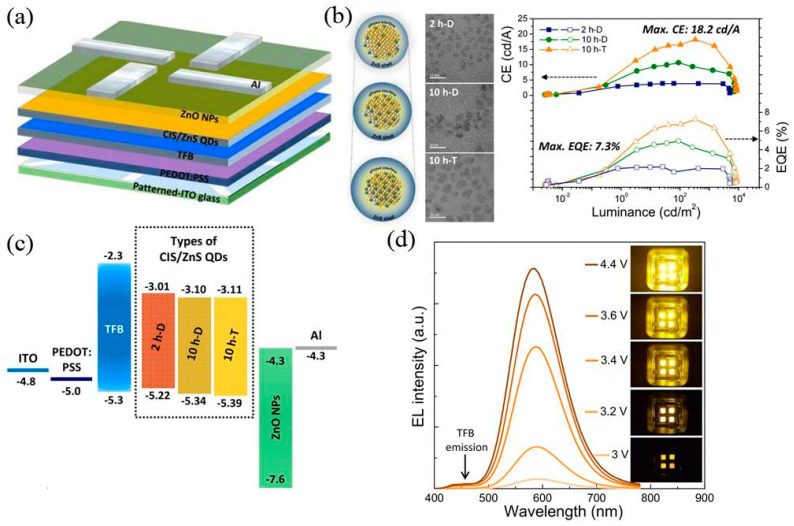
(**a**) Device schematic, (**b**) current efficiency and external quantum efficiency as a function of luminance, (**c**) corresponding energy level diagram and (**d**) EL spectra with increasing driving voltage of EL devices. Reprinted with permission from ref. [181]. Copyright 2016, American Chemical Society.

The surfaces of QDs are modified with long chain ligands (such as oleic acid and oleamine), which hinders the charge injection between the transport layer and the emission layer, resulting in a low charge injection rate of the device, which seriously affects the luminous efficiency of the device. In addition, solvents used in the charge transport layer of EL devices are often similar to those used in the QD layer, leading to the decomposition of the luminescent layer and the limitation of the device structure [178]. To solve the above problems, Zhong et al. [179] proposed a simple in situ ligand exchange method to obtain alcohol-soluble CuInS_2_ QDs. They first synthesized olimine-modified CIS/ZnS QDs and then directly injected 6-mercaptohexanol (MCH) into the solution for an exchange reaction. Alcohol-soluble QDs are obtained by substituting the methyl end of the long chain oleamine with the hydroxyl end of the short chain MCH, which is then dissolved in polar solvents (such as methanol, ethanol, dimethylformamide, etc.). It is worth noting that the PLQY of QDs obtained by an in situ ligand exchange reaction is still 70.5% (cf. a PLQY of 72.3% before the reaction). Finally, they successfully applied the QDs as a luminescence layer in EL devices with a maximum luminance of 8735 cd/m^2^ and EQE of 3.22%. Yu et al. [180] pointed out that the short-chain ligand on the surface of QDs is conducive to reducing the migration distance of charge and the energy barrier between the charge transport layer and the luminescent layer. Meanwhile, it also enhances the charge injection efficiency between the interface of the charge transport layer and the luminescent layer of QDs, thus improving the photoelectric performance of the device. They replaced the long-chain ligand oleic acid on the surface of AgIn_5_S_8_/ZnS QDs with the short-chain ligand 1,2-ethyl dimercaptan, and applied it in ITO/ZnO/PEI/AIS/ZS/CBP/MoO_x_/Au EL devices, whose EQE increased from 0.026% to 1.52%. Yang et al. [181] obtained CIS/ZnS QDs with different shell thicknesses and interfacial alloying degrees by controlling the shell growth time, and used them as luminescent layers to prepare electroluminescent devices with an ITO/PEDOT:PSS/TFB/QDs/ZnO/Al structure (Figure 10). The study found that extending the coating time was conducive to obtaining CIS/ZnS QDs with a higher alloying degree. Using ZnS as a luminescent layer, they had a more stable interfacial potential. Meanwhile, the thicker ZnS shell could effectively inhibit the non-radiative transition processes, such as Auger recombination and Förster resonance energy transfer, and significantly improve the radiative recombination efficiency of excitons in the luminescent layer in EL devices. The luminance of the device was up to 8464 cd/m^2^, the current efficiency was 18.2 cd/A and the EQE was 7.3%.

## 6. Conclusions

In the past decades, II-VI (CdSe, ZnS), III-V (InAs, InP) and IV-VI type (PbS, PbTe) binary QDs have attracted attention due to their excellent optical properties. However, most of them contain toxic ions, which restricts their commercial applications. Therefore, the preparation of environmentally friendly QD materials with excellent optical performance has become a current hot issue. The I-III-VI type QDs (such as CuInS_2_, CuInSe_2_, AgInS_2_, AgInSe_2_, etc.) have excellent physical and chemical properties (e.g., strong absorption, broad excitation, wide and symmetrical emission peaks, controllable fluorescence size, good photobleaching stability and high quantum yield). Meanwhile, their optical properties can be tuned by controlling their shape, size, composition and surface functionalization, and thus have great application potential in the fields of solar cells, light-emitting diodes and electroluminescence devices, etc. At present, the preparation of multiple QDs can be divided into two ways, i.e., organic phase synthesis and aqueous phase synthesis. The photoluminescence intensity, luminescence range and Stokes shift of multiple QDs can be effectively improved by doping metal ions (Zn^2+^, Mn^2+^ and Cu^+^) or surface coating. However, there are still some important scientific and technical problems that need to be solved in the preparation and application of I-III-VI type QDs: (1) The mechanism related to Cu doping and its oxidation state in the QDs is still controversial. Two valence states may exist after Cu doping into QDs, i.e., the Cu^+^ and Cu^2+^ oxidation states. Therefore, how to establish the mechanism of Cu doping QDs and determine the oxidation state of Cu have become the primary problems to be solved for Cu-based multiple QDs. (2) By changing the dominant factors such as dopant concentration and preparation method, the doped QDs appear as single emission or multiple emission. However, the emission spectrum of co-doped QDs is difficult to regulate delicately, and the photoluminescence intensity of QDs is still low due to the various ions involved in the synthesis process. Therefore, the surface defect emission is very sensitive to the surrounding environment. The preparation and the interaction mechanism of I-III-VI type QDs with a high PLQY co-doped by transition metal ions have become the main trend for the development of QDs [182]. (3) When the concentration of QDs is higher or converted from colloid to powder, the aggregation-induced effect leads to an increase in QD size and a fluorescence quenching phenomenon, resulting in a decrease in the luminous efficiency of the assembled WLED devices. Moreover, the insolubility between the organic ligand on the surface of QDs and the polymer matrix and the aging of the matrix due to the heat of the chip cause the optical conversion efficiency to inevitably decreases [183]. Therefore, it is still a challenge to select suitable ligands and substrates to obtain high performance WLEDs. (4) The parameters of EQE, LE and luminance of EL devices are still far behind those of cadmium and perovskite QD-based EL devices. It is necessary to further study the working mechanism of the devices and rationally design the structure of each functional layer in order to achieve a charge injection balance. In conclusion, the development of I-III-VI type QDs with high performance and stability has a profound impact on scientific research and commercial applications.

## Figures and Tables

**Table 1:** Organic preparation methods for I-III-VI type QDs.

Materials	Precursors, Ligands, Solvents	Methods	Conditions	Emission Peak/nm	Size/nm	PLQY/%	Ref.
Cu-In-S/ZnS	CuI, In(AC)_3_, Zn(St)_2_, S, DDT, OAm, ODE	Hot injection	130 °C, N_2_	530~642	2.3~4.8	85	[71]
Ag-In-S/ZnS	AgNO_3_, In(AC)_3_, Zn(Ac)_2_, S, DDT, OA, OAm, TOP, ODE	Hot injection	130 °C N_2_	603~868	4.9	72	[72]
Ag-Cu-Ga-Se/ZnSe	AgI, CuI, Ga(acac)_3_, ZnI_2_, Se, DDT, OAm, ODE	One-pot	120 °C N_2_	510~620	4.45~5.78	71.9	[7]
Cu-In-S/ZnS	CuI, In(AC)_3_, Zn(Ac)_2_, DDT, OA, ODE	Solvothermal	180 °C	651~775	4.37	85.06	[73]
Cu-Zn-Ga-Se/ZnSe	CuI, Ga(acac)_3_, Se, ZnI_2_, DDT, OAm, ODE	One-pot	120 °C, N_2_	509~624	3.01~3.93	72.6	[74]
Ag-In-Zn-S/ZnS	AgNO_3_, In(AC)_3_, Zn(Ac)_2_, S, DDT, OAm, ODE	Heating up	180 °C, N_2_	475~645	3.36~4.65	-	[64]
Cu-In-S	Cu(Hfacac)_2_·xH_2_O, InCl_3_, Nadedtc·3H_2_O, DDT, ODE	Hot injection	140 °C, Ar	568~630	3.8~5.6	-	[75]
Ag-In-Zn-S	AgNO_3_, In(Ac)_3_, DDT, OA, OAm, ODE	Hot injection	110 °C	550~635	3.4~5	60.3	[65]
Ag-In-Zn-S	AgNO_3_, InCl_3_, Zn(St)_2_, S, DDT, OAm, OCA, ODE	Hot injection	180 °C, Ar	625~720	4.5~8.8	67	[76]
CdSe/Ag-Zn-S	CdO, Se, AgNO_3_, ZnO, S, Myristic acid, TOP, TOPO, HDA, OA, ODE	Hot injection	240 °C, Ar	588~623	4.1~10.2	~100	[77]

**Table 2:** Aqueous phase preparations and PLQY of I-III-VI type QDs.

Materials	Precursors, Ligands	Methods	Conditions	Emission Peak/nm	Size/nm	PLQY/%	Ref.
Ag-In-S/ZnS	AgNO_3_, InCl_3_, Na_2_S·9H_2_O, Zn(Ac)_2_, MAA	Heating up	90~95 °C	580~770	2.0~3.5	47	[88]
Ag-In-S/ZnS	AgNO_3_, In(OH)_3_, Zn(NO_3_)_2_·6H_2_O, (NH_4_)_2_S, TGA, Gelatin, PVA	Electric pressure cooker	120 °C	560~575	2.5~3.4	64	[89]
Ag-In-S	AgNO_3_, InCl_3_, PEI, Na_2_S·9H_2_O	Electric pressure cooker	120 °C	550~560	3.1	32	[90]
Cu-In-S/ZnS	CuCl_2_·2H_2_O, InCl_3_, Zn(Ac)_2_·2H_2_O, Thiourea, SC, GSH	Microwave	95 °C	543~700	3.2~4.8	43	[43]
Ag-In-Zn-S	C_2_H_3_O_2_Ag, In(NO_3_)_3_·4.5H_2_O, C_3_H_7_NO_2_S	Heating up	100 °C	550~650	1.8~2.5	30	[91]
Cu-In-Zn-S	CuCl_2_·2H_2_O, InCl_3_·4H_2_O, Zn(OAc)_2_·2H_2_O, GSH, SC, Na_2_S	Hot injection	95 °C	588~668	3.5~3.9	5.95	[62]
Ag-In-S	AgNO_3_, InCl_3_, GSH, citric acid, NH_4_OH, HNO_3_, Na_2_S·9H_2_O	Heating up	96~98 °C	-	1.5~1.7	30.5	[92]
Ag-In-S	C_2_H_3_O_2_Ag, In(NO_3_)_3_·4.5H_2_O, C_3_H_7_NO_2_S	Heating up	80~100 °C	475~665	3.0~5.3	-	[93]
Ag-In-S	AgNO_3_, InCl_3_, GSH, citric acid, NH_4_OH, Na_2_S·9H_2_O, (NH_4_)_2_S	Continuous flow chemistry	80~150 °C	588~668	1.1~2.3	44	[94]
Ag-In-S	AgNO_3_, InCl_3_, GSH, citric acid, NH_4_OH, Na_2_S·9H_2_O	Heating up	96~98 °C	-	0.5~1.2	30	[95]
Ag-In-S	AgNO_3_, InCl_3_, L-cysteine	Biomineralization	37 °C	550~605	1.6~2.2	7.8	[96]

## Data Availability

The data that support the findings of this study are available from the corresponding author upon reasonable request.
